# Patents for Surgical Instruments

**Published:** 1865-06

**Authors:** 


ARTICLE XXII.

### PATENTS FOR SURGICAL INSTRUMENTS.

Mr. Editor:—It seems to me that it is time for the profession to take measures to stamp with its strong disapprobation the practice of members of our fraternity taking out patents for surgical instruments. I see in the Transactions of the National Association, and of the New York State Medical Society, articles accepted and published from two men who have been notoriously guilty of disregarding the professional etiquette in regard to this matter—men who accept and use all the discoveries of the rest of the profession, which have been given them free and without cost, and then having devised something themselves, they, with a meanness which is despicable, refuse to reciprocate, but declare that no professional brother shall be allowed to make use of their inventions without paying them for the patent right. This pernicious example is being followed, to some extent, even at the West.

These men ask, Why should not a surgeon be protected in his inventions, as well as a mechanic? Perhaps a few remarks on this subject may serve to set the matter in a clearer light in the minds of those who ask the question.

Abstractly speaking, there is no immorality in a physician or a surgeon owning a patent, but practically there is an inexpediency in it. For, in the first place, it is unseemly. The physician and surgeon have to deal with human life, and the sacredness of that interest is such, that the whole profession, and the whole world, have agreed in the sentiment that the attempt to limit the use of any life-saving discovery is barbarous. It is something as though some clergyman should try to patent, for his own benefit, any plan capable of promoting the moral and religious welfare of mankind. Moved by this feeling, the profession, the world over, have worked with all their genius and energy, to make medical and surgical improvements, and each man has magnanimously thrown his results into the common free stock. Now the attitude of any surgeon who patents an instrument, is this:—He has received, free of cost, the right to use all the medicines and instruments which have been devised by the profession in the whole world; he uses them daily; if he is, in the least degree, an intelligent man, he practices all the while on ideas furnished him, without charge, by his brethren in all parts of the world; and then, accepting all these, he ungratefully refuses to reciprocate the courtesy, but patents his own petty devices, hoping to profit somewhat by preventing those who have furnished him all these advantages from receiving anything in return. It is fair to say that this is unmitigated meanness, and it is perfectly proper to exclude such men from the benefits of professional fellowship, since they despise its established courtesies.

There is another sting to their selfishness. Surgeons generally derive a majority of their business from the courtesy of other practitioners, who refer surgical cases to them. This is especially true of orthopedic surgeons, who are the chief sinners in the matter of patents. Now, to use all the discoveries of others free, to receive half their income from patients sent to them by the profession, and then to refuse the small quota of their own inventions to the very men who feed them, is contemptible. If there were any very great advantages to be gained by the patent, the meanness would be more excusable; but a patent surgical instrument scarcely ever pays the cost of production to a practitioner. The only way for a man to make such a thing pay, is to abandon his practice and take up the trade of instrument maker. He will then no longer be a surgeon, but a mechanic, and, as such, at liberty to patent his devices. The real way for a surgeon to make money, is to throw his improvements freely and magnanimously before the world for use, and reap the reputation which is so readily given to those who make valuable discoveries. This reputation always draws in tenfold more profit, by the increase of his business, than can ever be pinched out of the community by means of patent rights. I hope the National Association will take this matter up and bring the weight of its influence to prevent the further degradation of American Surgery in this respect. X.

